# Observations on the Bloody Murrain of Cattle

**Published:** 1841-10

**Authors:** John Winans

**Affiliations:** Green County, Ohio


Art. III.—*Observations on the Bloody Murrain of Cattle.* By John Winans, M. D., of Green County, Ohio.

From the formidable mortality occasioned by this disease among the cattle of this region, it has lately become a subject of interest. Formerly nobody paid much attention to it, except those who suffered from its influence. Now, however, the attention of the medical and agricultural community has become awakened to the importance of trying to discover the cause, that they may be enabled to prevent it, and thus save from destruction a vast amount of property.

What number of our cattle die from this disease, I am unable from any data in my possession to determine. Judging however from some observations in a section of the country devoted entirely to the raising of stock, I should say that the mortality annually is one in every twenty-five. Some seasons it is much greater. One man in our neighborhood, who generally keeps about one hundred head on hand, says that in one season he lost twenty-five.

Cattle of all ages do not seem equally liable to the disease. Sucking-calves do not have it at all; and it has seldom been observed in animals under one year old.

All observers agree that it seldom, if ever, occurs in animals that are *lean.* Its attacks are confined exclusively to cattle in good order, and fat cattle. So universally is this the case, that fatness is considered by some as a predisposing cause—by some as an indispensable condition of the system.

No period of the year is exempt from the disease, though its greatest ravages are made in the fall months. Males and females seem equally liable to it.

*Symptoms.*—Loss of appetite is generally the first manifestation of ill health. This is soon followed by thirst, and a contracted appearance of the whole body. This however may be common to it and other disorders. But infallibly manifesting the presence of the disease, are the *discharges of blood from the fæcal and urinary passages.* Often but little, and sometimes no blood makes its appearance in the dejections from the bowels. The urinary organs on the contrary keep up a perpetual discharge of blood and urine, amounting sometimes to three or four gallons in twenty-four hours.

*Prognosis.*—This is universally bad. Few if any cases are known to have recovered after the disease had got complete possession of the system. Copious discharges of blood from the bladder are always regarded as fatal; for farmers, when they discover this, seldom make a remedial effort, but abandon the animal to its fate.

*Cause.*—Of the predisposing or exciting causes, nothing is certainly known. The proximate cause or pathology of the disease itself is, I think, now known, and consists in *inflammation of the kidneys*, which, if not arrested, terminates in *gangrene and death.* With a number of causes assigned for this disease by stock-growers, among the most popular of which was that of leeches (*flukes*) in the liver, I lately went to work and made a post-mortem examination of an animal that had died of a well-marked case of the disease.

The animal, a cow, four years old, and in fine order, was discovered to be unwell about a week previous to her death. The day before she died she discharged a quantity of blood from the anus and bladder—most copious from the latter.

On opening the thorax the right lung was discovered to be in a slightly inflamed condition. The thorax contained half a pint or more of blood and water.

Attention was next directed to the liver, where I expected to find leeches, (*flukes.*) Examining this organ carefully, I could discover none, nor any traces of primary disease. In the adipose matter surrounding the kidney I noticed a red discoloration denoting previous inflammation. Removing the upper portion developed it more intensely; and on exposing the right kidney it was found to be in a complete state of mortification, incapable of sustaining its own weight. The left was similarly affected though not so much softened and disorganized. The color of both was black, though the right was much the most so.

The bowels in the vicinity of the kidneys were much more inflamed than any where else, and that portion passing over the right kidney and terminating in the rectum, had its mucous surface inflamed, and it contained a quantity of the coloring matter of the blood in the form of paste.

The other organs of both abdomen and chest, appeared to have suffered from nothing but sympathy and juxtaposition. Externally there were some spots of ecchymosis, and the peritoneum had evidently suffered considerable inflammation.

The bladder contained half a gallon of fluid blood, which did not coagulate on cooling, and the mucous surface of the organ was inflamed.

The contents of the large and small intestines were but little altered from the natural healthy appearance, except that portion of them extending from the kidneys to the anus, which contained, as stated above, a quantity of the coloring matter of the blood of the consistence of paste.

*Treatment.*—According to these pathological views, the treatment of the disease should consist in *blood-letting*, and other antiphlogistic means.

*October*, 1840.

